# PON-P2: Prediction Method for Fast and Reliable Identification of Harmful Variants

**DOI:** 10.1371/journal.pone.0117380

**Published:** 2015-02-03

**Authors:** Abhishek Niroula, Siddhaling Urolagin, Mauno Vihinen

**Affiliations:** Department of Experimental Medical Science, Lund University, Lund, Sweden; Universita’ di Padova, ITALY

## Abstract

More reliable and faster prediction methods are needed to interpret enormous amounts of data generated by sequencing and genome projects. We have developed a new computational tool, PON-P2, for classification of amino acid substitutions in human proteins. The method is a machine learning-based classifier and groups the variants into pathogenic, neutral and unknown classes, on the basis of random forest probability score. PON-P2 is trained using pathogenic and neutral variants obtained from VariBench, a database for benchmark variation datasets. PON-P2 utilizes information about evolutionary conservation of sequences, physical and biochemical properties of amino acids, GO annotations and if available, functional annotations of variation sites. Extensive feature selection was performed to identify 8 informative features among altogether 622 features. PON-P2 consistently showed superior performance in comparison to existing state-of-the-art tools. In 10-fold cross-validation test, its accuracy and MCC are 0.90 and 0.80, respectively, and in the independent test, they are 0.86 and 0.71, respectively. The coverage of PON-P2 is 61.7% in the 10-fold cross-validation and 62.1% in the test dataset. PON-P2 is a powerful tool for screening harmful variants and for ranking and prioritizing experimental characterization. It is very fast making it capable of analyzing large variant datasets. PON-P2 is freely available at http://structure.bmc.lu.se/PON-P2/.

## Introduction

Rapidly advancing high-throughput sequencing technologies produce enormous amounts of genomic data. The increasing speed and decreasing cost of sequencing paves way for exome- and complete genome-based personalized medicine [1]. The major challenge to use genomics in personalized medicine is the same as in genetic diagnosis, namely the interpretation of effects and impacts of genetic variants [2].

Computational approaches are essential for screening harmful variations as the huge amounts of generated sequence data are practically impossible to analyze using experimental methods. For example, the Database for Short Genetic Variations (dbSNP) build 138 (released April 2013) [3] contains over 62 million human variants which is about 9 million more than in the previous build released 10 months earlier. Similarly, Catalogue Of Somatic Mutations In Cancer (COSMIC) v66 (released July 2013) [4] consists of more than 600,000 somatic single nucleotide variations (SNVs) leading to amino acid substitutions. The 1000 Genomes Project estimated that each individual carries 10,000–11,000 non-synonymous variations in addition to 10,000–12,000 synonymous variations in the coding regions [5]. These numbers are small in comparison to the variations in non-coding regions, but the coding variants are more frequently associated in diseases than non-coding variants [6]. Non-synonymous SNVs causing amino acid substitutions are the most common variations associated with Mendelian diseases.

Several computational tools have been developed to predict whether variations are deleterious. These methods are based on different principles and assumptions including features derived from evolutionary conservation, protein structure, sequence environment, functional annotations, and physical and biochemical properties of amino acids. SIFT [7] and PROVEAN [8] are entirely dependent on evolutionary conservation while other tools such as MutPred [9], PolyPhen-2 [10], SNAP [11], and SNPs&GO [12] utilize combination of evolutionary conservation and other types of features. Condel [13] and PON-P [14] are meta-predictors and use outputs of other tools as input to make consensus prediction. Although different methods based on different training features and datasets are available, recent studies [15,16] indicate that currently available tools are sub-optimal, and more reliable tools are required to accurately predict the disease-relevance of the variants. An additional requirement is that the prediction methods have to be fast to cope with exome and complete genome sequencing datasets.

Features based on evolutionary conservation are powerful for classification of amino acid substitutions as demonstrated by the results of evolutionary conservation based approaches including PANTHER [17], PROVEAN [8] and SIFT [7]. Most currently available methods use protein sequences and their homologs to represent evolutionary conservation while some methods have adopted a phylogenetic approach by using selective pressure as evolutionary information [18,19]. Highly conserved sequence positions are likely functionally and/or structurally important and are assumed to be under strong selective pressure during evolution [20]. Variations at such sites reduce the fitness of the carriers, which are selected against, and the individuals are likely to be removed from the population. A powerful way to represent selective pressure is by calculating the ratio of non-synonymous (Ka) to synonymous substitution rates (Ks). Previous studies [18,19] show that selective pressure at codon-level is useful for discriminating disease related variants from neutral ones. However, to our knowledge, it has not been implemented to available tools as it is computationally intensive to calculate codon-level Ka/Ks ratio [12].

Most prediction methods score variants and classify them into two classes, pathogenic and neutral separated by a threshold score. Variants that obtain a score close to threshold are less reliably predicted because even a small change could move them from one class to another. In pattern recognition, classification with reject option has been used to classify examples with reliable scores and to remove cases with unreliable scores [21]. By optimizing thresholds, the error rate of a classifier is minimized as ambiguous and unreliable predictions are removed [22]. This option is highly applicable in variation classification because the phenotype of individuals having the same variation may vary widely depending on several factors such as environmental exposure, age, status of immune system, medical history, etc.

Here, we describe a novel computational tool, PON-P2, for variant tolerance prediction. It classifies amino acid substitutions into three categories–pathogenic, neutral or unknown tolerance. PON-P is a meta-predictor which uses the predictions of other methods as features while PON-P2 is a novel tool that employs features of evolutionary sequence conservation, properties of amino acids, GO annotations and functional annotations, if available. PON-P2 is a machine learning-based tool trained and tested on benchmark datasets. It shows consistently improved performance when compared with the state-of-the-art tools. Evaluation of prediction time showed that it is significantly faster than other methods, thereby being able to analyze exome and genome wide datasets for identifying potentially harmful variants.

## Materials and Methods

### Dataset

Benchmark variation data was downloaded from VariBench [23], a database for variation datasets and consisted of 14,610 pathogenic and 17,393 neutral variations. A subset of the dataset containing 14,086 pathogenic variants in 1,082 proteins and 14,848 neutral variants in 6,598 proteins, for which all the features used in PON-P2 (excluding functional annotations) were available, was used for training and testing PON-P2. The dataset was divided into two parts i) one-tenth of the data was used as test dataset; ii) the remaining nine-tenths were used for feature selection and training. The dataset was divided in such a way that proteins in the same family were either in test or training dataset. The proteins were mapped to the protein families in Pfam database (Pfam 27.0) [24]. The training and test datasets are publicly available in VariBench (http://structure.bmc.lu.se/VariBench/tolerance.php) along with Variation Ontology (VariO) annotations [25].

### Features

**Amino acid features**. AAindex [26] contains three databases for altogether 685 physicochemical and biochemical properties of amino acids. 617 features were used after eliminating those with missing values. The features in AAindex1 have a numerical index for each amino acid while those in AAindex2 and AAindex3 are amino acid substitution matrices. For each variant, the difference between the indices for the reference and variant amino acid were calculated for AAindex1 features while the values were taken directly from AAindex2 and AAindex3 matrices.

**Gene Ontology feature**. The GO terms derived features have previously been used in variant classification [12,27]. The GO terms associated with each protein were extracted from UniProtKB/Swiss-Prot. All the ancestors for each GO term were collected with R bioconductor tool GO.db (http://www.bioconductor.org/packages/2.13/data/annotation/html/GO.db.html). The GO terms were then filtered so that each protein had each term only once. Two separate sets of GO terms were created for each class (pathogenic and neutral). The summation of log ratio of the frequency of GO term in the pathogenic set to the frequency of GO term in the neutral set is calculated as:

$$LR=\sum \log\frac{f(P_{i})+1}{f(N_{i})+1}$$ (1)

Where *LR* is the GO feature value for a protein; *f*(*P*_*i*_) and *f*(*N*_*i*_) are the frequencies of the i^th^ GO term in pathogenic and neutral datasets, respectively. To avoid undetermined ratios, 1 was added to the frequencies.

**Evolutionary conservation features**. The ratio of non-synonymous substitution rate to synonymous substitution rate (ω) estimates selective pressure. Conserved sites are often structurally or functionally crucial and variations at such sites may be unfavorable. Synonymous variations are more common than non-synonymous variations and thus ω is higher for variable sites than for conserved sites. Orthologous protein and cDNA sequences for each human protein (translated from the longest transcript) were collected from Ensembl compara database [28] using perl application program interface (API). The orthologous protein sequences were aligned with ClustalW [29]. Based on the protein multiple sequence alignment, the codon alignment of cDNA sequences was generated using PAL2NAL [30]. The cDNA codon alignment was provided for selecton [31] to calculate codon-level ω. The human sequence was used as the reference sequence and the number of iterations was set to 1. Besides ω, other features that represent sequence profile including the proportions of reference and variant amino acids, and the number of sequences in the protein sequence alignment were used.

**Functional and structural annotations**. Site specific annotations were determined from UniProtKB/Swiss-Prot and PDB. The variations which occur at such sites were identified. The distribution of the annotations in the pathogenic and neutral datasets were calculated. The annotations, for which proportion of variations in either class was greater than 0.85, were selected.

### Feature selection

The feature selection was performed in two steps. We combined two greedy feature selection approaches–backward elimination and forward selection [32]. In the first step, 10 feature subsets were selected by backward elimination method one from each 10-fold cross-validation set. The 10 feature subsets selected in the first step were combined together and a forward feature selection was performed in the second step. In the forward feature selection, the performance of each feature was evaluated by 10-fold cross-validation. The training data was split into 10 parts so that all variants in one protein family were strictly present in one of the partitions. 9 partitions were used for training and the remaining partition was used for testing. The first feature selection step included the following procedures:

1. A random forest classifier was trained using all 622 features.
2. The accuracy of the classifier was measured by using the cross-validation testing dataset and the features were ranked based on mean decrease in gini index.
3. The feature that obtained the least mean decrease in gini index was eliminated.
4. Another random forest classifier was trained using the remaining features.
5. Steps 2 to 4 were repeated until there was only one feature left.
6. The accuracies of all the classifiers were compared and the features, used in the classifier with the highest accuracy, were selected.

In the second step, we performed a forward feature selection to select the features that improve the performance by highest margin. First, a *non-redundant feature set* with all the features in the 10 subsets (from first feature selection) was obtained. An empty feature subset was initiated and was called *selected feature set*.

1. A random forest classifier was trained using *non-redundant feature set*. The features were ranked by using random forest mean decrease in gini index. The highest ranked feature was added to *selected feature set* and eliminated from *non-redundant feature set*.
2. Another classifier was trained by using features in *selected feature set* and the accuracy was measured.
3. Features in *selected feature set* and one feature from *non-redundant feature set* was used to train a classifier and the accuracy was measured.
4. Step 3 was iterated for all the features in *non-redundant feature set*.
5. The feature that improved the accuracy by highest percentage was added to *selected feature set* and eliminated from *non-redundant feature set*.
6. Steps 3, 4 and 5 were repeated until no improvement was achieved by addition of any of the features.

Then, the *selected feature set* was used to train PON-P2.

### Random forest

PON-P2 uses randomForest package which is an R interface to the original random forest algorithm [33]. The number of features used to generate random feature subset was set to default value of 2. By stratified random sampling with replacement, 200 bootstrap samples, containing the same number of cases as the original training data, were generated and a classifier was trained on each bootstrap sample. The number of trees grown in each random forest was set to 300 as the prediction of random forest was reported to be stable at 300 when increasing the number of trees [14].

### Using functional annotation information

The probability of pathogenicity for a variation occurring at a functionally annotated site is estimated from the probability predicted by random forest and proportion of variations (annotated as occurring in functional sites) in pathogenic class using following rule

$$P_{c}(p)=P_{a}(p)+P_{rf}(p)-P_{a}(p)\times P_{rf}(p)$$ (2)

where, *P*_*c*_(*p*) is the combined probability of pathogenicity for the variation; *P*_*a*_(*p*) is the probability of variation to be pathogenic, which is derived from the proportion of pathogenic variations in training dataset for the annotation type and *P*_*rf*_ (*p*) is the probability of pathogenicity of the variation predicted by random forest.

### Determining the reliability

The variations predicted with high confidence are identified by using probabilistic method. Although we cannot determine the probability distribution function of bootstrap probabilities, we can apply Chebysev’s inequality as it is applicable to any arbitrary distribution. For a random variable X with mean μ and standard deviation σ, Chebysev’s inequality guarantees that at least 1-(1/k^2^) values lie within k standard deviations from mean

$$P(\mu -k\sigma <X<\mu +k\sigma )\geq 1-\frac{1}{k^{2}}$$ (3)

While 1-(1/k^2^) is 0.95, if range of μ±*k*σ excludes 0.5, the prediction is labeled as reliable and is classified as either pathogenic or neutral. Otherwise, the variation is reported as unclassified.

### Performance evaluation

The performance of PON-P2 and other prediction methods were evaluated by using six measures as recommended for binary classifiers [34,35]. The measures include positive predictive value (PPV), negative predictive value (NPV), sensitivity, specificity, accuracy and Matthews correlation coefficient (MCC). These measures are defined mathematically as follows:

$$PPV=\frac{TP}{TP+FP}$$ (4)

$$NPV=\frac{TN}{TN+FN}$$ (5)

$$Sensitivity=\frac{TP}{TP+FN}$$ (6)

$$Specificity=\frac{TN}{TN+FP}$$ (7)

$$Accuracy=\frac{TP+TN}{TP+TN+FP+FN}$$ (8)

$$MCC=\frac{TP\times TN-FP\times FN}{\sqrt{\left(TP+FP)(TP+FN)(TN+FP)(TN+FN\right)}}$$ (9)

where, TP and TN are the number of correctly predicted pathogenic and neutral cases, respectively, and FN and FP are the number of incorrectly predicted pathogenic and neutral cases, respectively.

Performance cuboids were used to visualize the six major performance scores simultaneously in a 3-dimensional space. The overall performance measure (OPM) of a classifier is represented by normalized volume of the performance cuboid, which ranges from 0 to 1. Normalized MCC (nMCC) is calculated by rescaling the value of MCC from 0 to 1. The performance cuboids were obtained by plotting the six performance scores using rgl package (http://cran.r-project.org/web/packages/rgl/index.html) in R.

$$nMCC=\frac{1+MCC}{2}$$ (10)

$$OPM=\frac{\left(PPV+NPV)(Sensitivity+Specificity)(Accuracy+nMCC\right)}{8}$$ (11)

## Results

### Feature selection and classifier design

**Fig 1:**
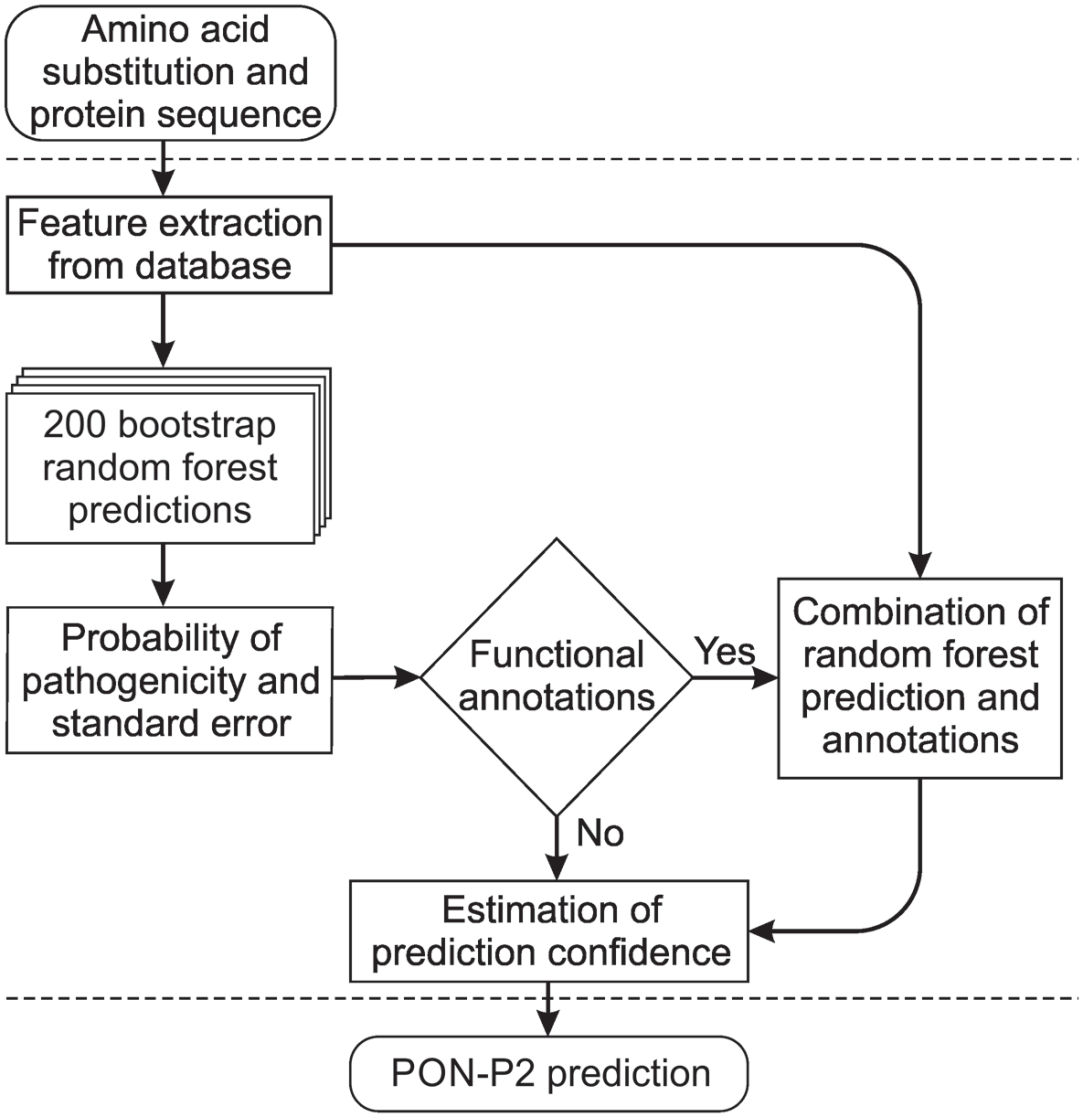
Overview of PON-P2 architecture and implementation. PON-P2 uses pre-calculated feature vectors and bootstrap random forest for prediction. In addition, it makes benefit of information about functional and/or structural annotations, when available, and identifies reliably predicted variations and groups them either as pathogenic or neutral.

PON-P2 is a random forest predictor for pathogenicity-association of amino acid substitutions (Fig. 1). It is trained on annotated disease-causing variants as positive cases and variants with allele frequency > 0.01 in dbSNP as neutral cases. Extensive feature selection was performed to identify useful features for discrimination of disease-related variants from neutral ones. Eight useful features were selected from 622 features. The selected features were GO annotations, codon-level Ka/Ks, 3 features representing sequence profile and 3 physical and biochemical properties of amino acids including KOSJ950114 [36], RACS820113 [37] and TANS770104 [38] (S1 Table). From 10 rounds of feature selection, 5 features (GO, frequency of reference amino acid, KOSJ950114, Ka/Ks, number of sequences in MSA) were overlapping in all 10 selected feature sets.

**Fig 2:**
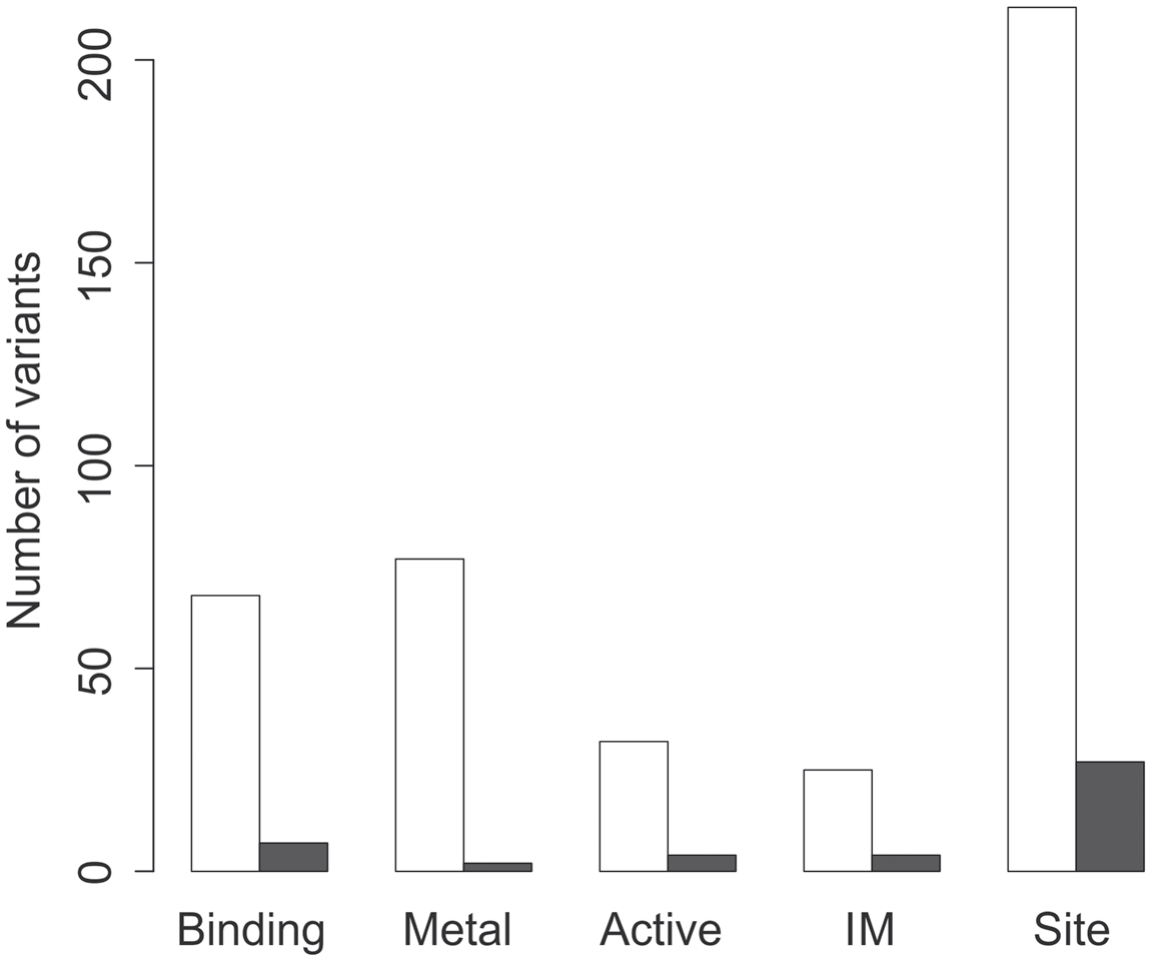
Distribution of variations at functional and structural sites. The pathogenic variations are represented by white bars and neutral variations by grey bars. The functional and structural annotation sites were obtained from Swiss-Prot and PDB. Binding, binding site; Metal, metal binding site; Active, active site; IM, intra membrane region; Site, catalytic, co-factor, anti-codon, regulatory or other essential site surrounding ligands in the structure.

Random forest algorithm ranks the features based on mean decrease in gini index. A fraction of the training data is used to train a classifier and the remaining part is used to estimate the decrease in gini index [33]. The higher the decrease in gini index, the more important is the feature. The GO derived feature has high importance while the amino acid features are less important (S1 Table). The annotations of variations to functional and structural sites were collected from UniProtKB/Swiss-Prot and Protein Data Bank (PDB) (S2 Table). The distributions of variations in the pathogenic and the neutral datasets were computed to examine the disease-relation of variations at functional and structural sites. Five types of functional annotations were selected for which the proportion of variation in either class was greater than 0.85 (Fig. 2). This bias towards one class was utilized as additional information for the predictor. If a variation occurs at a site with functional annotations, the probability of pathogenicity of a variation at the functional site is combined with random forest probability to make final prediction (Fig. 1).

### Performance of feature subsets

**Fig 3:**
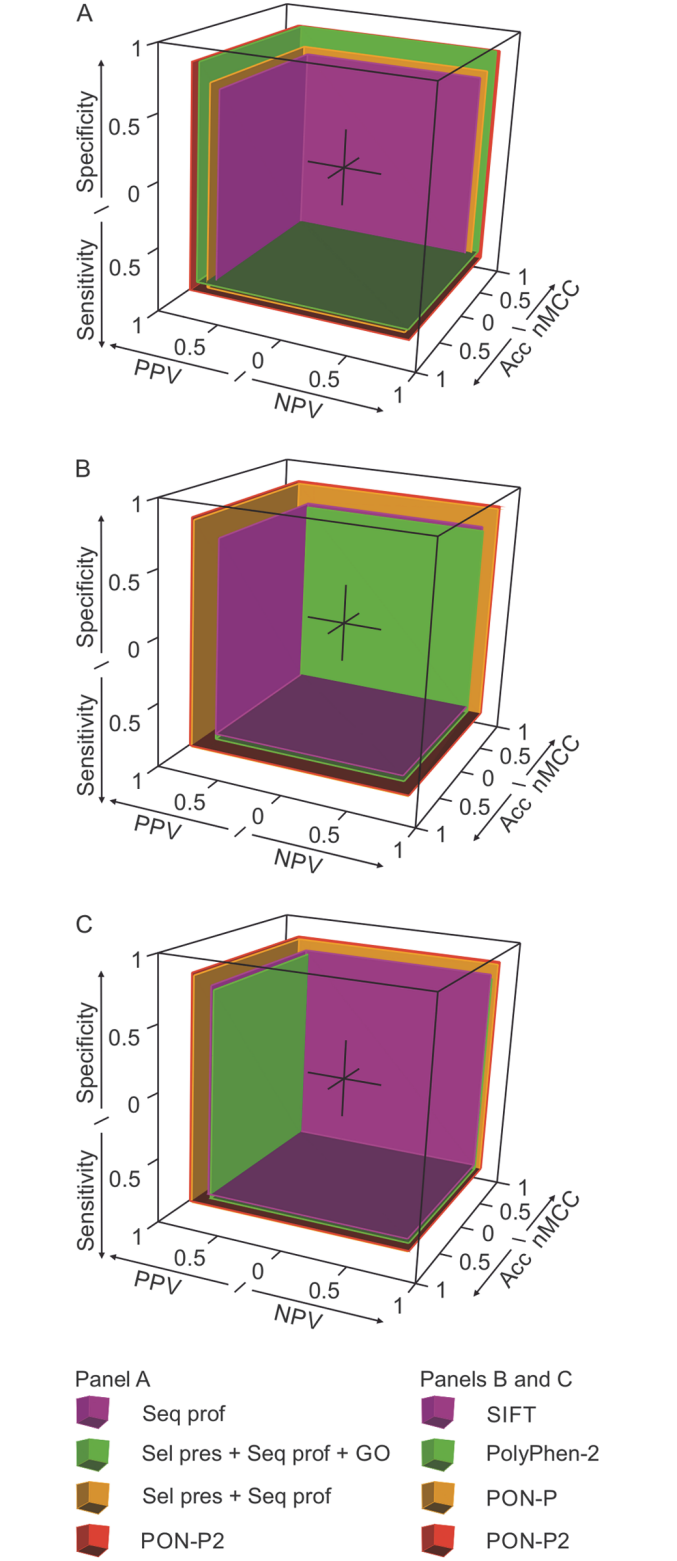
Performance cuboids for PON-P2 and other methods. Six performance measures: PPV, NPV, sensitivity, specificity, acc (accuracy) and normalized MCC (nMCC = MCC×0.5+0.5) for each method are represented by the distances of the six faces of the cuboid from the origin. (A) Performance cuboids for different feature subsets used in PON-P2. Seq prof, Proportions of reference and altered amino acids and number of sequences in multiple sequence alignment; Sel pres + Seq prof, evolutionary features; Sel pres + Seq prof + GO, evolutionary features and GO annotations (B) Performance cuboids for PolyPhen-2, PON-P, PON-P2 and SIFT for all predicted variations by each method on independent test dataset. The performance scores for PON-P and PON-P2 are for predictions at 0.95 confidence level. OPMs for PolyPhen-2, PON-P, PON-P2 and SIFT are 0.41, 0.61, 0.63 and 0.40, respectively. (C) Performance cuboids for predictors using c95-test set. OPMs for PolyPhen-2, PON-P, PON-P2 and SIFT are 0.47, 0.61, 0.63 and 0.48, respectively.

To estimate the contribution of each feature subset, we used combinations of features to train random forest classifiers and compared their performance on the test dataset. Sequence profile showed higher performance than selective pressure, and combination of sequence profile and selective pressure further improved the performance as well as the proportion of predicted variants (Table 1). Evolutionary conservation features show slightly lower performance but higher coverage than GO annotations and amino acid features together. Evolutionary conservation features perform even better when combined with GO derived feature and amino acid features (Fig. 3A). Although the performance contributions of individual features are small, the performance evaluation shows that each feature subset contributes to the performance of PON-P2 and elimination of any of the features results in poorer performance (Table 1).

**Table 1:** Prediction performance of feature subsets on test data.

	PPV^a^	NPV^a^	Sens^a^ ^,^ ^b^	Spec^a^ ^,^ ^b^	Acc^a^ ^,^ ^b^	MCC^a^	OPM^a^ ^,^ ^b^	Coverage^c^
AA^b^	0.65	0.77	0.74	0.68	0.71	0.42	0.36	0.63
SeqProf^b^	0.67	0.79	0.80	0.66	0.73	0.46	0.39	0.74
SelPres^b^ + SeqProf	0.72	0.83	0.84	0.71	0.77	0.55	0.46	0.53
AA + GO^b^	0.73	0.82	0.71	0.84	0.79	0.55	0.47	0.37
SeqProf + GO	0.78	0.84	0.74	0.87	0.82	0.62	0.53	0.51
AA + SelPres	0.81	0.85	0.83	0.83	0.83	0.66	0.57	0.45
AA + SeqProf	0.82	0.83	0.80	0.85	0.83	0.65	0.56	0.49
AA + SelPres + SeqProf	0.82	0.85	0.81	0.85	0.83	0.67	0.58	0.52
SelPres + SeqProf + GO	0.78	0.87	0.79	0.86	0.83	0.65	0.56	0.53
AA + SeqProf + GO	0.82	0.87	0.81	0.88	0.85	0.69	0.61	0.57
AA + SelPres + GO	0.82	0.89	0.86	0.86	0.86	0.71	0.63	0.52
PON-P2	0.82	0.89	0.85	0.86	0.86	0.71	0.63	0.62

^a^All scores are calculated for the variations that were predicted at confidence level 0.95.

^b^Sens, Sensitivity; Spec, Specificity; Acc, Accuracy; OPM, Overall performance measure; AA, Amino acid features; GO, GO annotation derived feature; SelPres, Selective pressure; SeqProf, Sequence profile features (proportion of reference amino acid, proportion of variant amino acid and number of sequences in the multiple sequence alignment)

^c^Coverage is the proportion of the data that are predicted either pathogenic or neutral.

Performance improvement by using annotations of functional and structural sites was estimated. A significant number of variants at functionally annotated sites were predicted with unreliable score by using the random forest. After combining the annotation information and random forest prediction, the rejection rate decreased considerably, however, with comparable accuracy (S3 Table). As the number of variants at functionally annotated sites is small, the contribution to the overall performance scores is relatively small; however, it is large for the variants at functionally annotated sites.

### Benchmarking PON-P2

PON-P2 was tested by using 10-fold cross-validation and an independent test dataset. In the 10-fold cross-validation, variants in the same protein and protein family were strictly placed in either training or test set. The accuracy, MCC and OPM of PON-P2 at confidence level 0.95 were 0.90, 0.80, and 0.73, respectively for 10-fold cross-validation and 0.86, 0.71 and 0.63, respectively, for the test dataset. PON-P2 showed highest performance scores when compared with other methods. The performance scores are higher than for the other tools even when the unreliable cases were classified as pathogenic or neutral based on the predicted probability (cutoff 0.5) (Table 2). An independent analysis of bioinformatics tools for variations in Usherin protein showed that PON-P2 had the highest sensitivity (0.95) and specificity (0.98) among the predicted cases [39]. GO annotation is protein based feature which is the same for pathogenic and neutral variants in a protein. To test the discriminative power of PON-P2 for pathogenic and neutral variants in the same protein, we retrieved amino acid substitutions from dbSNP with allele frequency > 0.01 for those proteins that contain pathogenic variants in the test data. 382 variants were identified in 62 proteins. Among 192 variants predicted with high confidence, 162 (84.4%) were classified as neutral by PON-P2. Thus, PON-P2 is not overfitted and it classifies both pathogenic and neutral variants correct in the same protein.

**Table 2:** Performance scores of different prediction methods.

	Condel	PPH2^a^	Provean	SIFT	SNAP	PON-P^b^	PON-P2^b^ ^,^ ^c^
10-fold cross-validation							
TP	8626	10387	10170	8928	10140	6432	6375 (10191)
TN	7820	7960	9189	8577	8763	5787	7860 (10572)
FP	2894	4042	3887	3708	4299	993	805 (2497)
FN	2566	2182	2469	2451	3420	880	778 (2396)
PPV	0.75	0.72	0.72	0.71	0.70	0.87	0.89 (0.80)
NPV	0.75	0.79	0.79	0.78	0.78	0.87	0.91 (0.82)
Sens^d^	0.77	0.83	0.81	0.79	0.81	0.88	0.89 (0.81)
Spec^d^	0.73	0.66	0.70	0.70	0.67	0.85	0.91 (0.81)
Acc^d^	0.75	0.75	0.75	0.74	0.74	0.87	0.90 (0.81)
MCC	0.50	0.50	0.51	0.48	0.48	0.73	0.80 (0.62)
OPM^d^	0.42	0.42	0.43	0.41	0.41	0.65	0.73 (0.53)
Independent test data set							
TP	852	952	870	869	1077	567	638 (969)
TN	972	975	1135	1062	1092	722	909 (1255)
FP	353	470	432	432	513	137	144 (350)
FN	266	230	312	259	224	96	113 (332)
PPV	0.71	0.67	0.67	0.67	0.68	0.81	0.82 (0.74)
NPV	0.79	0.81	0.78	0.80	0.83	0.88	0.89 (0.79)
Sens	0.76	0.81	0.74	0.77	0.83	0.86	0.85 (0.75)
Spec	0.73	0.68	0.72	0.71	0.68	0.84	0.86 (0.78)
Acc	0.75	0.73	0.73	0.74	0.75	0.85	0.86 (0.77)
MCC	0.49	0.48	0.46	0.48	0.51	0.69	0.71 (0.53)
OPM	0.42	0.41	0.39	0.40	0.43	0.61	0.63 (0.45)

^a^HumVar trained PolyPhen-2. The performance of this version was better than for HumDiv trained PolyPhen-2 (data not shown).

^b^Performance scores are computed by using the predicted variants at 0.95 confidence level.

^c^Performance scores inside parentheses are for the predictor when the unreliable cases are included.

^d^Sens, Sensitivity; Spec, Specificity; Acc, Accuracy; OPM, Overall performance measure.

PON-P and PON-P2 reject the unreliable cases and classify the cases that are reliable at confidence level 0.95. To make a comparison of the performance of the methods using the same set of variants, we filtered out the variants rejected by PON-P2 and called the set of remaining variants c95-training and c95-test sets. c95-training set contains 61.7% of the training data while c95-test set contains 62.1% of the test data. The performance scores for all the methods (except PON-P as it automatically rejects unreliable cases) were computed. The methods show somewhat higher performance scores for both c95-training and c95-test sets. However, the other methods have still clearly lower performance than PON-P2 (S4 Table). These results show that rejection of the unreliable cases improves prediction performance significantly for all the methods. The performance scores for the different methods indicate that PON-P2 is the most balanced method in regards to the six performance scores. The real differences in performance (Fig. 3B,3C) are even larger as some of the methods have the benefit of being trained with cases in our dataset.

Recently, new predictors including MutationTaster2 [40] and Combined Annotation Dependent Depletion (CADD) [41] have been released. Because the tools have limited batch submission options, we compared the performance on the MutationTaster2 test dataset from http://www.mutationtaster.org/info/Comparison_20130328_with_results_ClinVar.html. We excluded the variations that were present in PON-P2 training dataset. The number of benign variants is higher than the number of deleterious variations, so we performed random sampling to select the same number of neutral and deleterious variations. The accuracy and MCC of PON-P2 were 0.95 and 0.90, respectively, which are higher than those for the other methods. The performance of PON-P2 and Mutation Taster2 were comparable when unreliable cases were predicted as pathogenic or neutral based on the predicted probabilities (cutoff 0.5) (Table 3). Although this data seems to be biased as indicated by the data provider, the performance of the methods is still comparable as the results are biased on the same direction for all the methods.

**Table 3:** Performance scores of prediction methods on data used by MutationTaster2 dataset.

	CADD^a^ ^,^ ^b^	Condel	PPH2^c^	Provean	SIFT	MT2^a^	PON-P2^d^
TP	503	439	506	507	530	548	327 (501)
TN	541	541	543	540	525	523	363 (571)
FP	59	59	57	60	75	77	1 (29)
FN	97	161	94	93	70	52	37 (99)
PPV	0.90	0.88	0.90	0.89	0.88	0.88	0.98 (0.95)
NPV	0.85	0.77	0.85	0.85	0.88	0.91	0.91 (0.85)
Sens^a^	0.84	0.73	0.84	0.85	0.88	0.91	0.90 (0.84)
Spec^a^	0.90	0.90	0.91	0.90	0.88	0.87	1.00 (0.95)
Acc^a^	0.87	0.82	0.87	0.87	0.88	0.89	0.95 (0.89)
MCC	0.74	0.64	0.75	0.75	0.76	0.79	0.90 (0.79)
OPM^a^	0.66	0.55	0.67	0.66	0.68	0.71	0.85 (0.72)

^a^CADD, Combined Annotation Dependent Depletion; MT2, MutationTaster2; OPM, Overall performance measure; Sens, Sensitivity; Spec, Specificity; Acc, Accuracy

^b^Variants with C-score greater than 15 were considered as deleterious and lower than 15 were considered as neutral as suggested by the method developers.

^c^HumVar trained PolyPhen-2. The performance of this version was better than for HumDiv trained PolyPhen-2 (data not shown).

^d^Performance scores are computed by using the predicted variants at 0.95 confidence level. The scores in the parentheses are for the predictor when the unreliable cases are included.

Most other tolerance prediction methods do not use classification with reject option. To check whether the performance of PON-P2 has improved only by rejecting the unreliable cases, we classified all the variants into binary classes. The probability cutoff of 0.5 was used above which the variants were predicted as pathogenic. The performance scores on such a binary classification showed that PON-P2 performs the best even when the unreliable cases are included (Tables 2 and 3). Hence, it clearly shows that the performance improvement is not solely due to the reject option but because of the robustness of the tool. SNAP was designed for predicting the functional effects of the variations and not optimized for prediction of disease-related variants. So, the performance comparison of SNAP with PON-P2 may not be optimal although SNAP has been widely used for pathogenicity prediction.

### Performance cuboid and overall performance measure

In machine learning, Receiver Operating Characteristic (ROC) curve and area under the ROC curve (AUC, also called for AROC) have been widely used to evaluate the performance of binary classifiers. A ROC curve shows the relative trade-off between true positive rate (TPR) and false positive rate (FPR) when different thresholds are set to distinguish between the two classes [42]. Classifiers like PON-P2, that are optimized to predict discrete classes, produce only a single point in the ROC curve thus being uninformative. For comprehending the full performance of a classifier, use of six performance measures has been recommended [35].

For the visualization and comparison of method performance, a novel projection to 3-dimensional space was developed. Assuming that a cube centered at origin represents the performance of a perfect classifier, the six major performance scores are represented by the distance of six faces of the cube from the origin. The performance scores of an imperfect classifier do not always produce a cube. Hence, we name the visualization method as performance cuboid. The overall performance of a predictor is estimated by calculating the volume of the cuboid and normalizing it from 0 to 1, referred to as overall performance measure (OPM).

Fig. 3 visualizes the comparison of different classifiers using performance cuboids. Only three faces of the cuboids are shown in full for better visibility. The classifier that gains the lowest performance scores is the closest to the origin i.e. has the smallest volume. The best performing predictor has its faces furthest away from the origin. For example, in Fig. 3B, SIFT and PolyPhen-2 achieve the lowest performance score and PON-P2 achieves the highest score. Therefore, the faces of the cuboids for SIFT and PolyPhen-2 are more visible while only small portion of the faces of the cuboid for PON-P2 are visible. The balanced overall performance of the predictor is given by OPM. OPMs for SIFT, PolyPhen-2, PON-P and PON-P2 are 0.41, 0.42, 0.65, and 0.73, respectively (Fig. 3B). The visualization and OPM scores show that PON-P2 performs better than the other predictors.

### Prediction time

With increasing amounts of genomic data and increasing possibility of personalized medicine, it is clearly evident that fast computational tools are a necessity for identification of deleterious variations. PON-P2 utilizes computationally expensive features like codon-level selective pressure to improve the performance of classifier. Computing the feature vector takes longer time than the prediction. To allow fast run times, we collected the protein sequences (translated from the longest transcripts) for all the coding human genes in Ensembl database [43] and computed the feature vectors for each position in these sequences and stored in a relational database. When a user submits a query, PON-P2 extracts the feature vectors from the database and runs the prediction. Hence, the time required for making sequence alignment and preparing the feature values is skipped. The time required by PON-P2 and some other methods to complete a typical prediction task was compared. PON-P2 is significantly faster than any other method (S5 Table). The result shows that PON-P2 is capable of handling the huge amounts of genomic variation data generated by modern sequencing technologies.

### PON-P2 web application

PON-P2 web application is freely available at http://structure.bmc.lu.se/PON-P2/. It has a user-friendly web interface. It accepts variations in multiple formats. Identifier submission requires for amino acid substitution(s) and one of UniProtKB/Swiss-Prot accession id, ensembl gene identifier or entrez gene identifier. When using gene identifiers, the variations have to be in the longest isoform of the gene. PON-P2 maps the UniProtKB/Swiss-Prot protein and entrez gene identifiers to the longest transcript of the corresponding gene in ensembl. Genomic submission is for nucleotide variations with chromosome number and location. PON-P2 accepts also genomic submissions in Variant Call Format (VCF), a widely used format to store the genomic variations in sequencing projects. For these submissions, PON-P2 makes predictions only for variations leading to amino acid substitutions. Sequence submission is for fasta format amino acid sequence and variations in it. Batch submission for all the submission formats is accepted and recommended. The results are sent to the user by email when ready.

## Discussion

The handling of immense amount of variation data generated by next-generation sequencing technologies and relating them to diseases is a major challenge. Several computational tools based on different principles have been developed to rank and prioritize non-synonymous SNVs for experimental characterization. However, currently available tools are sub-optimal [16] and are not capable for fast interpretation of the amount of data being generated. SIFT [7], PolyPhen-2 [10] and some other tools provide precalculated scores and predictions for all possible variations in large number of human proteins. Therefore, these methods provide predictions faster if the precalculated predictions are used. However, our analysis showed that the performance of these methods is lower than for PON-P2 and some other existing methods. Hence, the need of more reliable and faster computational tools persists. To address the requirement we have developed a novel tool, PON-P2. It is based on evolutionary conservation, structural and functional annotations and properties of amino acids and predicts whether a variation is harmful or not.

PON-P2 is trained on approximately equal numbers of disease-causing variations (positive dataset) and variations being relatively frequent (allele frequency > 0.01) in dbSNP (neutral dataset). Although the proportions of the harmful and benign variants in human are unknown, the best performance of binary classifiers are obtained by training with balanced dataset regardless of the composition of the true data [44]. The positive dataset was collected from databases and checked manually or automatically to be annotated as disease-causing. We feel that this provides the best starting point for developing variation tolerance predictor. Information about functional effects of variations have been used to train some other predictors. A problem emerges with such datasets because the functional effects are vaguely described e.g. in the widely used Protein Mutation Database (PMD) [45]. Secondly, there is not usually information about the biological effect. There is for example an extreme case of adenosine deaminase activity in severe combined immunodeficiency (SCID) where activity of 0.11% is sufficient for normal phenotype [46]. On the other hand, very minor change in activity (increase or decrease) can be harmful in other cases. Thus, changes in protein activity level are not necessarily sufficient to explain functional effects of variations.

We performed extensive feature selection to identify useful and non-redundant features. 8 features were selected from among 622 features. The attributes selected in PON-P2 are physical and biochemical properties of amino acids, GO and functional annotation, selective pressure and sequence profile. In a previous study, selective pressure together with sequence profile was observed to be more efficient than using them separately for classifying variants [19]. The analysis was performed with a comparatively small dataset consisting of about 11,000 variants. We evaluated the contribution of selective pressure and sequence profile using a more comprehensive variation dataset consisting of 28,934 variants. Both the selective pressure and the sequence profile improve the performance of classifier when combined with amino acid features and improves the prediction coverage when they are used together (Table 1). The contribution of amino acid features, GO annotations, and conservation features were evaluated and elimination of any of these feature subsets decreases the performance of the predictor (Fig. 3A). Only 3 out of the 617 features in AAindex turned out to be useful for the prediction. These include one substitution matrix (KOSJ950114) and two protein structural features (RACS820113 and TANS770104). Thus, although AAindex mainly contains simple amino acid propensities, they are uninformative.

Using classification with reject option reduces the error rate of a classifier by making predictions only for reliable cases [21,22]. We use Chebysev’s inequality and bootstrap method to determine the reliability of prediction. Using Chebysev’s inequality, if the predicted probability is reliable at confidence level 0.95, the variation is classified as pathogenic or neutral. Otherwise, the variant is designated as unclassified. Increasing the confidence level further reduces the error rate but on the other hand, the rejection rate also increases. Therefore, we optimized the method at confidence level 0.95 where the error rate is comparatively low and a significant fraction of variants (62.1%) can be classified as pathogenic or neutral. The concept of classification with reject option has not previously been used in tolerance predictions apart from PON-P. The concept is relevant in tolerance prediction because the genetic variants cannot always be classified distinctly into pathogenic or neutral groups. There are variants with intermediate effects which may be deleterious or neutral depending on other parameters. The same variant, even in monozygotic twins, can cause different phenotype [47], thereby excluding the simple binary classification scheme utilized in most of the other predictors. Thus, it is essential to identify unreliable predictions and reject them to reduce the false predictions. This is further evidenced by the improvement in the performance scores of all the compared methods when excluding the unreliable cases identified by PON-P2 (S4 Table). The superior performance scores for PON-P2 when all the variants are predicted into binary classes indicate that the performance improvement is not solely due to the reject option but because the method is robust.

Although codon-level selective pressure was observed to improve the discrimination of disease-related variations from neutral [18,19], it has not been employed previously in prediction methods probably because of being computationally intensive. We computed all features including selective pressure for each position in proteins (translated from the longest transcript) of all coding human genes and stored in a database. Despite the fact that PON-P2 uses bootstrap method, that requires more computation time for prediction, PON-P2 is significantly faster than the other methods (S5 Table). The speed is essential for interpretation of variants in large scale sequencing projects e. g. for application to personalized medicine.

The human genome is not completely annotated. Therefore, some of the features used in PON-P2 may be unattainable for some variants. For example, GO feature cannot be calculated if there are no GO annotations for a protein. In such cases, PON-P2 provides prediction based on other selected features except GO. The selective pressure and sequence profile features are based on multiple sequence alignments of ortholog sequences. If the sequence is unique for human, PON-P2 does not make predictions as it would not be reliable.

PON-P2 is capable of predicting variation effects in 86% of human proteins with high accuracy. PON-P2 has both improved prediction performance and computation time, thus making it suitable for ranking, prioritizing and filtering of large scale variation datasets.

## Supporting Information

S1 Table Importance scores of features used in PON-P2. (DOCX)

S2 Table List of functional and structural sites collected from UniProtKB/Swiss-Prot and PDB. (DOCX)

S3 Table Performance contribution of annotation features after combining with random forest prediction results in 10-fold cross-validation. (DOCX)

S4 Table Performance scores for c95-training and c95-test sets. (DOCX)

S5 Table Estimation of prediction time. (DOCX)
