# Supplementary material for: PON-P2: Prediction Method for Fast and Reliable Identification of Harmful Variants
Source: PLoS One. 2015 Feb 3;10(2):e0117380. doi: 10.1371/journal.pone.0117380 (PMC4315405; doi:10.1371/journal.pone.0117380)
Supplement: S1 Table — (DOCX) [file pone.0117380.s001.docx]

**Table S1. Importance scores of features used in PON-P2.**

| **Name** | **Importance score (SD)^a^** | **Description** |
| --- | --- | --- |
| GO annotation | 3191 (53.0) | Summation of log odd ratios of GO terms |
| **Evolutionary conservation features** | | |
| Selective pressure | 2678 (77.4) | Ratio of rate of non-synonymous substitutions to rate of synonymous substitutions (ω=Ka/Ks) |
| Proportion of reference amino acid | 1726 (62.34) | Ratio of frequency of reference amino acid to total number of sequences at variation site in the multiple sequence alignment |
| Number of sequences | 1342 (26.4) | Total number of sequences at variation site in multiple sequence alignment |
| Proportion of variant amino acid | 885 (46.0) | Ratio of frequency of variant amino acid to total number of sequences at variation site in the multiple sequence alignment |
| **Amino acid features** | | |
| KOSJ950114^b^ | 975 (23.3) | Context-dependent optimal substitution matrices for buried residues |
| RACS820113^b^ | 789 (13.0) | Value of theta (i) |
| TANS770104^b^ | 784 (12.6) | Normalized frequency of chain reversal R |

^a^SD; Standard deviation. The importance score is the average of importance scores for 200 bootstrap random forests. In random forest package, the importance score is calculated by adding the decrease in gini for each variable over all trees in the forest.

^b^AAindex abbreviations
