# Supplementary material for: PON-P2: Prediction Method for Fast and Reliable Identification of Harmful Variants
Source: PLoS One. 2015 Feb 3;10(2):e0117380. doi: 10.1371/journal.pone.0117380 (PMC4315405; doi:10.1371/journal.pone.0117380)
Supplement: S2 Table — (DOCX) [file pone.0117380.s002.docx]

**Table S2. List of functional and structural sites collected from UniProtKB/Swiss-Prot and PDB.**

| **Annotations** | **Description** |
| --- | --- |
| **NON_STD^a^** | Occurrence of non-standard amino acids (selenocysteine and pyrrolysine) in the protein sequence |
| **TRANSMEM^a^** | Extent of a membrane-spanning region |
| **BINDING^a^** | Binding site for any chemical group (co-enzyme, prosthetic group, etc.) |
| **SIGNAL^a^** | Sequence targeting proteins to the secretory pathway or periplasmic space |
| **METAL^a^** | Binding site for a metal ion |
| **ACT_SITE^a^** | Amino acid(s) directly involved in the activity of an enzyme |
| **CROSSLNK^a^** | Residues participating in covalent linkage(s) between proteins |
| **INTRAMEM^a^** | Extent of a region located in a membrane without crossing it |
| **SITE^b^** | Amino acid residues comprising catalytic, co-factor, anti-codon, regulatory or other essential sites or environments surrounding ligands present in the structure |
| **CISPEP^b^** | Prolines and other peptides found to be in the cis conformation |
| **SSBOND^b^** | Amino acid residues involved in disulfide bond |
| **LINK^b^** | The LINK records specify connectivity between residues that is not implied by the primary structure. |

^a^Annotations were extracted from UniProtKB/Swiss-Prot.

^b^Annotations were extracted from PDB.
