# Supplementary material for: PON-P2: Prediction Method for Fast and Reliable Identification of Harmful Variants
Source: PLoS One. 2015 Feb 3;10(2):e0117380. doi: 10.1371/journal.pone.0117380 (PMC4315405; doi:10.1371/journal.pone.0117380)
Supplement: S3 Table — (DOCX) [file pone.0117380.s003.docx]

**Table S3. Performance contribution of annotation features after combining with random forest prediction results in 10-fold cross-validation.**

| **Method** | **TP** | **TN** | **FP** | **FN** | **UV^a^** | **Total** | **Coverage^b^** | **Accuracy^c^** |
| --- | --- | --- | --- | --- | --- | --- | --- | --- |
| **Random forest** | 222 | 20 | 4 | 4 | 144 | 394 | 0.63 | 0.97 |
| **Random forest combined with annotations** | 288 | 19 | 12 | 4 | 71 | 394 | 0.82 | 0.95 |

^a^UV, number of unclassified variations

^b^Proportion of variants predicted either pathogenic or neutral.

^c^Only variations that occur at functional sites were considered.
