# Supplementary material for: PON-P2: Prediction Method for Fast and Reliable Identification of Harmful Variants
Source: PLoS One. 2015 Feb 3;10(2):e0117380. doi: 10.1371/journal.pone.0117380 (PMC4315405; doi:10.1371/journal.pone.0117380)
Supplement: S4 Table — (DOCX) [file pone.0117380.s004.docx]

**Table S4. Performance scores for c95-training and c95-test sets.**

|  | **Condel** | **PPH2^a^** | **Provean** | **SIFT** | **SNAP** | **PON-P^b^** | **PON-P2** |
| --- | --- | --- | --- | --- | --- | --- | --- |
| **10-fold cross-validation** | | | | | | | |
| **TP** | 5327 | 6261 | 6193 | 5425 | 6082 | 6432 | 6375 |
| **TN** | 5672 | 5860 | 6570 | 6228 | 6166 | 5787 | 7860 |
| **FP** | 1511 | 2087 | 1906 | 1937 | 2496 | 993 | 805 |
| **FN** | 1021 | 880 | 945 | 1041 | 1055 | 880 | 778 |
| **PPV** | 0.78 | 0.75 | 0.76 | 0.74 | 0.71 | 0.87 | 0.89 |
| **NPV** | 0.85 | 0.87 | 0.87 | 0.86 | 0.85 | 0.87 | 0.91 |
| **Sens^c^** | 0.84 | 0.88 | 0.87 | 0.84 | 0.85 | 0.88 | 0.89 |
| **Spec^c^** | 0.79 | 0.74 | 0.78 | 0.76 | 0.71 | 0.85 | 0.91 |
| **Acc^c^** | 0.81 | 0.80 | 0.82 | 0.80 | 0.78 | 0.87 | 0.90 |
| **MCC** | 0.63 | 0.62 | 0.64 | 0.60 | 0.56 | 0.73 | 0.80 |
| **OPM^c^** | 0.54 | 0.53 | 0.55 | 0.51 | 0.47 | 0.65 | 0.73 |
| **Independent test data set** | | | | | | | |
| **TP** | 540 | 544 | 500 | 545 | 628 | 567 | 638 |
| **TN** | 689 | 705 | 800 | 759 | 742 | 722 | 909 |
| **FP** | 186 | 248 | 231 | 228 | 311 | 137 | 144 |
| **FN** | 134 | 111 | 155 | 135 | 123 | 96 | 113 |
| **PPV** | 0.74 | 0.69 | 0.68 | 0.71 | 0.67 | 0.81 | 0.82 |
| **NPV** | 0.84 | 0.86 | 0.84 | 0.85 | 0.86 | 0.88 | 0.89 |
| **Sens** | 0.80 | 0.83 | 0.76 | 0.80 | 0.84 | 0.86 | 0.85 |
| **Spec** | 0.79 | 0.74 | 0.78 | 0.77 | 0.70 | 0.84 | 0.86 |
| **Acc** | 0.79 | 0.78 | 0.77 | 0.78 | 0.76 | 0.85 | 0.86 |
| **MCC** | 0.58 | 0.56 | 0.53 | 0.56 | 0.53 | 0.69 | 0.71 |
| **OPM** | 0.50 | 0.47 | 0.45 | 0.48 | 0.45 | 0.61 | 0.63 |

^a^HumVar trained PolyPhen-2 was used. The performance of this version was better than for HumDiv trained PolyPhen-2 (data not shown).

^b^The method rejects unreliable data automatically, so all cases, from entire dataset, predicted at 0.95 confidence level were included.

^c^Sens, Sensitivity; Spec, Specificity; Acc, Accuracy; OPM, Overall performance measure.
