# Supplementary material for: PON-P2: Prediction Method for Fast and Reliable Identification of Harmful Variants
Source: PLoS One. 2015 Feb 3;10(2):e0117380. doi: 10.1371/journal.pone.0117380 (PMC4315405; doi:10.1371/journal.pone.0117380)
Supplement: S5 Table — (DOCX) [file pone.0117380.s005.docx]

**Table S5. Estimation of prediction time.**

| **Prediction method** | **Number of proteins** | **Number of variants** | **Time required^a^** | **Processor information** |
| --- | --- | --- | --- | --- |
| **PolyPhen-2^b^** | 7331 | 28934 | 58 minutes | Intel Core i7-2600 @ 3.4 GHz |
| **SNAP^b^** | 7331 | 28934 | 2 weeks | 7 × Intel Core i7-2600 @ 3.4 GHz |
| **PON-P2** | 7331 | 28934 | 10 minutes | Intel Xeon E5-2665 @ 2.4 GHz |

^a^Prediction times are real times required by the tools for a batch submission.

^b^Standalone version was used in a single thread and precomputed multiple sequence alignments were used.
